# Risk Factors for Non-Adherence to Medication for Liver Transplant Patients: An Umbrella Review

**DOI:** 10.3390/jcm13082348

**Published:** 2024-04-18

**Authors:** Jordi Colmenero, Mikel Gastaca, Laura Martínez-Alarcón, Cristina Soria, Esther Lázaro, Inmaculada Plasencia

**Affiliations:** 1 Liver Transplant Unit, Hospital Clínic, IDIBAPS, CIBERehd, University of Barcelona, 08007 Barcelona, Spain; jcolme@clinic.cat; 2 Hepatobiliary Surgery and Liver Transplantation Unit, Biobizkaia Health Research Institute, Cruces University Hospital, University of the Basque Country, 48940 Bilbao, Spain; 3 Transplant Unit, Surgery Service, IMIB-Virgen de la Arrixaca University Hospital, 30120 Murcia, Spain; lma5@um.es; 4 Health Psychology, Suportias, 28806 Madrid, Spain; cristina.soria@suportias.com; 5 Faculty of Health Sciences, Valencian International University, 46002 Valencia, Spain; 6 Pharmacy Unit of the University Hospital of Nuestra Señora de Candelaria, 38010 Tenerife, Spain; iplagar@gobiernodecanarias.org

**Keywords:** liver transplantation, adherence, non-adherence, umbrella review, immunosuppressant medication

## Abstract

**Background/Objectives**: Liver Transplantation (LT) is the second most common solid organ transplantation. Medication adherence on LT patients is key to avoiding graft failure, mortality, and important quality of life losses. The aim of this study is to identify risk-factors for non-adherence to treatment of liver transplant patients according to reliable published evidence. **Methods**: An umbrella review within the context of adherence to immunosuppressant medication of LT patients, was conducted. The review was performed in accordance with the principles of the preferred reporting items for systematic reviews and meta-analysis (PRISMA) guidelines. **Results**: A total of 11 articles were finally included for the review. Non-adherence factors were identified and allocated using the WHO classification of factors for non-adherence. Each of these groups contains a subset of factors that have been shown to influence adherence to medication, directly or indirectly, according to literature findings. **Conclusions**: The results of the review indicate that sociodemographic factors, factors related to the patient, factors related to the treatment, condition-related and health system-related factors are good categories of predictors for both adherence and non-adherence to immunosuppressive medication in LT patients. This list of factors may help physicians in the treating and recognizing of patients with a potential risk of non-adherence and it could help in the designing of new tools to better understand non-adherence after LT and targeted interventions to promote adherence of LT patients.

## 1. Introduction

Liver Transplantation (LT) is estimated to be performed on roughly 6000–9800 individuals per year in Europe and the United States [1,2,3]. It is the second most common solid-organ transplantation in the world [4]. Hepatocellular carcinoma and chronic end-stage liver disease due to alcohol and metabolic-associated liver disease [5] are the most common indications for LT [6,7,8]. Biliary atresia is the most common indicator for LT in children [9]. One- and 3-year patient survival after LT have progressively improved over the past 20 years, yet long-term survival has only slightly increased. The main causes of long-term mortality are related to cardiovascular events, cancer and to a lesser extent graft failure. Despite the fact that graft failure due to rejection is uncommon after LT, it carries an increased risk of patient mortality and graft loss, especially in patients experiencing late rejection [10].

Current standard immunosuppression regimens in the early post-operative period frequently include a calcineurin inhibitor (mainly tacrolimus), an antimetabolite (mostly mycophenolate), and eventually corticosteroids [11]. On the other hand, beyond 3–6 months after LT most guidelines and consensus recommend either monotherapy with tacrolimus or anticalcineurin-sparing strategies using mycophenolate or everolimus [12]. In this scenario of anticalcineurin minimization, an adequate adherence to the immunosuppressive treatment is essential. Non-adherence to immunosuppression has been recognized to impair graft survival after LT [13].

LT is associated with significant physical and psychological symptoms [14] and side-effects which promote non-adherence. Thus, there is a growing need for understanding how to improve liver transplant patients’ health outcomes. In general, between approximately 50% and 60% of patients with chronic diseases demonstrate poor medication adherence (e.g., 27–40% of hypertension patients [15,16]; Up to 78% of renal transplant recipients [17]. The prevalence of non-adherence of LT patients is difficult to assess but the latest estimates vary between 3% and 47% according to a recent systematic literature review [12]. Non-adherence to the prescribed immunosuppression is associated with graft rejection, graft failure [18], contributing to 20% of late acute rejection episodes and 16% of graft losses within the overall transplant population [19] post-transplant mortality [10], poor health-related quality of life, and increased healthcare costs after LT [20,21,22,23,24]. The literature evidences that one in ten deaths of patients submitted to liver transplantation was related to non-adherence to the immunosuppressive medications [25]. Non-adherence is a particularly significant issue for adolescents and young LT patients, making the transition from pediatric to adult care a risky period for these patients, their families and their attending physicians [26].

Medication adherence is a dynamic and complex behavioral process and is intensely affected by individual, social, and environmental factors. The definition of medication non-adherence is wide [27,28], ranging from a deviation from the prescribed medication regimen sufficient to adversely influence the regimen’s intended effect [29], to eventual missing doses, extra doses, drug holidays, variable timing of intake, and poor understanding of the medication [12,28,30]. The World Health Organization (WHO) has provided a classification of factors that influence adherence [31]. The classification distinguishes between health system factors, socioeconomic factors, factors related to the treatment/therapy, patient related factors, and condition related factors. Most studies on medication non-adherence after solid-organ transplantation have focused mainly on kidney transplantation [32,33,34], whilst the extent and nature of medication-related problems among LT recipients are not that well-known [12]. 

In conclusion, non-adherence to prescribed medications is key to achieving adequate outcomes after LT and early identification of risk factors is essential. The main goal of our study was to perform an umbrella review of the literature aimed at identifying risk-factors for non-adherence to prescribed medication of LT. In addition, we also reviewed the bibliography on interventions to improve medication adherence of LT patients.

## 2. Materials and Methods

An umbrella review (a review of reviews and systematic literature reviews) within the context of adherence to immunosuppressant medication in LT, was conducted in September 2023. The review was not registered.

The review was performed in accordance with the principles of the preferred reporting items for systematic reviews and meta-analysis (PRISMA) guidelines [35]. The Population/Patient Intervention/Exposure Comparison Outcome (PICO/PECO) method was applied to structure the search [36]. The framework of this systematic review according to PICO was: Population: adult or adolescent population who have undergone LT; Intervention: treatment with immunosuppressant medication; Comparison: results from different reviews and new results compared to results from previous reviews; and Outcome: identification of risk factors for non-adherence of the targeted population to immunosuppressant medication.

### 2.1. Search Strategy

The search was conducted in the PubMed, Cochrane library of Systematic Literature Reviews, Web of Science and ProQuest scientific databases. This search was completed by a targeted search in Google Scholar, in order to manually retrieve some articles that were known to be relevant for the objective of this review, but that were not captured by the search algorithm at the scientific databases consulted. 

The search algorithm used was: “(adherence OR non-adherence OR nonadherence OR compliance OR noncompliance OR non-compliance) AND (transplant OR replacement) AND (hepatic OR liver)”. Word variations were searched. This search was limited to reviews, systematic reviews and meta-analyses, written in English, Spanish or French, published since 2010.

All abstracts captured by the search algorithm were downloaded to Zotero 6.0.22, the reference manager software used. In a first phase, abstracts and titles were reviewed to select the articles that met the criteria for full-text screening and data extraction.

### 2.2. Inclusion and Exclusion Criteria

The inclusion criteria were review articles of any type (including reviews, narrative reviews, overviews, systematic literature reviews) that had addressed completely or partially the risk factors for non-adherence of liver transplant in adult or adolescent patients. Articles were excluded if they were not review articles, if they focused only on children, or, articles addressing solid organ transplantation of multiple organs, if they only partially addressed the objective by reviewing one or a very small number of articles in LT. 

### 2.3. Data Extraction

For each article among those included for data extraction, the following information was extracted in an Excel template from the final selection of articles:

- General information (title, authors, journal, year of publication, abstract and country of study).
- Objectives: main and secondary (when there were) study objectives.
- Study design: type of review and description of methods and study selection criteria.
- Study of adherence post-LT: indicator of the study addressing completely/partially the issue of adherence within the context of LT, the number of included studies in the review (if available) that focused on risk factors, and the time horizon (when available); the type of patients (to distinguish between adolescent and adult populations, for example); adherence measures used; treatment regime (if specified: e.g., medication); adherence factors identified.
- Study setting/context: a field to identify the specific context of the article (e.g., LT in adolescents), and if the study controls or offers results according to the severity of the patients’ disease.
- Analysis: type of analysis conducted in the review (e.g., descriptive statistics), and if the review offers a comparison between population subgroups.
- Results: Main results regarding the adherence factors identified, and summary and discussion offered in the review.
- Conclusions: the main concluding remarks of the review article.
- Strong points and limitations of the review.

The original language of the texts was maintained to avoid a possible interpretation bias of the researchers in the data extraction phase.

### 2.4. Risk of Bias and Quality Assessment

The GRADE check-list was used for the assessment of certainty in the evidence provided by the texts reviewed [37]. The check-list was adapted to be able to correctly rate criteria according to the type of review conducted, respecting the overall structure and logic of the scale. Six criteria were rated and evaluated: 1—Unified statistical analysis on original data, 2—Confounders used, 3—Outcomes of interest pre-specified, 4—Consistency in the presentation of results (or heterogeneity adequately addressed if present), 5—Little likelihood of publication bias, and 6—Large sample (or a sufficient number of studies to conduct the review). Each domain was scored as 1 if the text was fulfilling the criteria and 0 otherwise. The overall certainty rating was computed as follows: “High” if none of the domains were rated 0, “Moderate” one domain was rated 0, “Low” and “Very low” if two or more domains were rated 0. Only publications of the highest quality (rated “high” or “moderate”) were included for the final synthesis of results.

For reviews that were not systematic, or for reviews that included multiple solid organ transplantation and not just LT, quality and bias were assessed by analysing the number of articles that were reviewed on LT. Studies that provided information based on a systematic literature review on the risk factors for non-adherence fully within the context of LT were considered to be the strongest. In addition, studies using control factors to provide results for specific subgroups, in addition to aggregate results (for example, on prevalence rates of non-adherence) were considered of higher quality and lower risk of bias. The number of studies the review included to extract results and conclusions regarding factors of influence for non-adherence was also an important criterion. It was considered that the greater this number, the greater the quality. 

### 2.5. Analysis Description

The analysis focuses on identifying the risk factors for non-adherence to medication regimes in order to achieve better success rates of LT. The focus of the analysis will be the population of adults or adolescents, excluding studies on children, given that it is the population that is most likely affected by serious liver diseases and requiring an LT. The review will also summarize the results regarding prevalence rates of non-adherence found in this specific context.

## 3. Results

### 3.1. Descriptive Results

**Figure 1:**
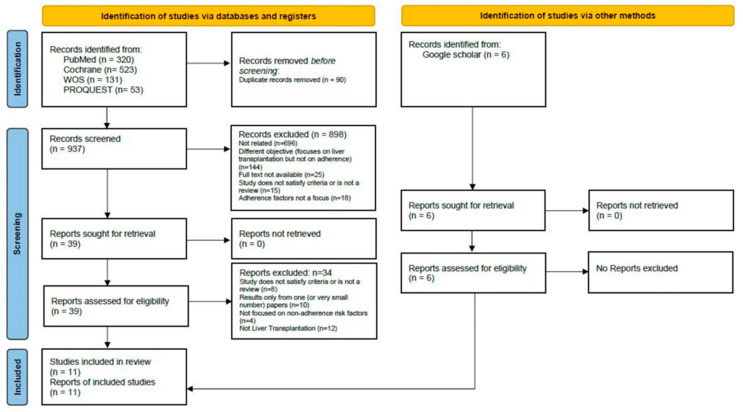
Prisma Flow chart.

In total, the search identified 1033 abstracts. Duplicates found (*n* = 90) were removed, leaving 943 abstracts for the final screening. The distribution of retrieved abstracts from the different databases consulted was: *n* = 320 abstracts from PubMed, *n* = 523 abstracts from Cochrane Systematic Reviews Library, *n* = 131 from WOS, *n* = 53 abstracts from ProQuest, and *n* = 6 abstracts from Google Scholar. In the end, 11 articles were included for full data extraction and analysis. The search and process of exclusion and papers’ selection are presented in the PRISMA flow diagram (Figure 1).

Results on the identification of adherence/non-adherence factors for LT recipients are shown in Table 1. This table also includes some descriptive information about the reviewed articles, including, for example, the population addressed in the paper or the results provided for prevalence rates of non-adherence. 

Among the 11 articles reviewed, four articles [39,40,41,42] did not provide results according to subgroups of age, but provided results for the overall population of LT recipients, five articles provided specific results on risk/enhancement factors for non-adherence/adherence for the adolescent population [12,13,14,38,44] and four articles reported similar factors, but for the adult population [12]. One article also provided specific results for living donor LT recipients [14]. 

Most papers (n = 9, 81.8%) were focused purely on LT, while the remaining two articles [39] included results for other types of solid organ transplantation (although they provided specific results on LT and those were based on a sufficient number of articles reviewed on such a population). Countries studied include North American countries (including the USA and Canada), France, Spain, Iran and India. Two papers were reviews for the North American and European countries [25,38]. Two articles were expert panel reviews [38,41], two were overviews [13,39], one article was a review of meta-analysis [40], one was an integrative review [25], one was a narrative review [12], two papers were reviews [12,14], one paper was a systematic literature review [44], and one paper was a brief review [42].

### 3.2. Adherence Measures Identified and Adherence Rate Findings

Medication (non)adherence (which in the context of LT is especially related to immunosuppressive medication adherence) was mentioned by all the papers included. Estimates and ranges are provided in Table 1. Variability is high, ranging from 3% to 80% when the age is not a controlled factor. However, some studies have shown adolescent non-adherence rates that are nearly 4 times higher when compared with adults [12].

Non-adherence to the medication regime prescribed is sometimes explained by behaviors (e.g., missing clinical appointments, taking extra doses or drug holiday) [12,13,41]. One study reported 17% of the LT recipients having medication trade-offs. Trade-offs included either reporting difficulty affording medications, spacing out medications, or making choices between buying medications and buying food, and were associated with a lower mean self-reported medication adherence (77% adherence with trade-offs compared to 89% without trade-offs) [13]. One study reported timing non-adherence rates (defined as not taking a dose at the prescribed time) that ranged from 27% to 64%, altered dose rates that ranged from 1% to 14%, and drug holiday rates that ranged from 0% to 39% [43]. Finally, the last review reports a clinical attendance non-adherence rate of 45% [45]. 

### 3.3. Factors Associated with (Non)Adherence for LT Patients

Factors were identified and allocated to one of the WHO groups of factors. All identified factors were considered good predictors for non-adherence in the reviewed literature, and are presented in Table 2. 

Related with the healthcare system, not having medical insurance or having Medicare insurance [13] were factors associated with medication non-adherence of LT patients. Particularly, Medicare insurance patients were more likely to report medication trade-offs. A study also found adherence improved with an intervention targeted at adherence in the adolescent population, showing that the lack of targeted interventions is also associated with poorer medication adherence outcomes for LT patients [14]. 

Among the socioeconomic factors, demographic characteristics were observed to have an influence over the non-adherence of LT patients. Being a male associated with medication non-adherence [25,42,43], also appeared to be a good predictor of missing doses [14]. Age also proved to be an influencing factor for non-adherence to medication, for older pediatric patients (adolescents) [42,44] and younger adults [12,43]. Being African American [43] was also identified as an influencing factor for medication non-adherence of LT patients. Being divorced was associated with non-adherence as per its association with higher missing doses [42], and in general with medication non-adherence [25,43]. Other identified social factors found to associate with medication non-adherence were: poor social functioning or social support instability [13,14,25,40,43] also found to be relevant before transplant [14,43], and found to be associated with higher missed doses [14], low income or financial barriers [13], higher education levels before transplantation [14], low literacy [13] and health literacy [43], being employed [43] and being unemployed at the time of listing for liver transplant [14,42,43], lower educational attainment [13], low family cohesion [44], being a single-parent family [44], living alone [13], autonomy from family, poor abstract thinking, understanding long-term consequences of present actions [38], lower conscientiousness before transplant [14]. 

Among patient-related factors influencing non-adherence of LT patients, the most commonly reported factors were: prior history of alcohol abuse, identified by two reviews [39,43], missing clinical appointments [42,43], and having an ongoing psychiatric illness, that also proved to be associated with missing clinical appointments [12,13]. Poor mental health also has a demonstrated association with non-adherence to medical regimes [25,44], with similar findings observed for patients having a pre-transplant diagnosis of mood disorder [14,42]. Mental health needs before transplantation were also found to be associated with non-adherence [43]. Being an active substance abuser [13,25] was also identified, by two reviews, to be associated with medication non-adherence, causing difficulties to comply with the recommended regimes or missing consultations. Other factors include prior history of non-adherence to medication [43], self-management skills, mainly the ability for functioning effectively in the adult healthcare system, meant for adolescents before transitioning to the adult life [38], having intact perspective memory [43], self-reported non-adherence before transplant [14], poor pre-operative adherence [42] and conviction that the medication is harmful [12].

In relation to treatment-related factors found to influence non-adherence of LT patients were: side effects of medications [12,13,14,43], high cost [12,13,14], difficult understanding of the medication regimes [13,14,41], the number of medications [14], lack of medication knowledge/poor medication understanding [13], lack of control and reduction of the number of doses [25], more immunosuppressant-related symptom frequency and/or symptoms before transplant [43].

Regarding condition-related factors, hospital readmission after transplantation was found associated with medication non-adherence [14]. A similar association was found with a higher number of comorbid conditions, [13] where the condition-related factors associated with medication non-adherence of LT patients was identified in the reviewed literature. A longer period of time from the transplant was also an identified factor associated with non-adherence [13,14,43].

In addition, one of the reviews [42] found a high risk for behavioral non-adherence is associated with having 4–6 of the following factors (moderate risk 2–3 factors; low risk 0–1 factor): 1. DSM-IV compliant diagnosis of mood or anxiety disorder within the 24 months prior to transplant; 2. passive versus active coping styles in the face of emotional stressors; 3. presence or absence of documented medication adherence issues prior to transplant; 4. presence or absence of DSM-IV diagnostic criteria for substance abuse/dependence prior to transplant; 5. presence or absence of a primary caregiver as social support for the transplant recipient; and 6. the presence or absence of documented concerns regarding the stability of that social support system.

### 3.4. Certainty of Evidence: GRADE Assessment Results

Results of the assessment of the evidence certainty are summarized in Table 3. All the included papers present high or moderate certainty of the evidence provided. The full data extraction template with detailed information to justify this assessment is available as Supplementary Materials (Table S1). Four of the eleven texts analyzed were rated as of “High” certainty of evidence [38,39,42,44], and the remaining seven texts were considered to provide “Moderate” certainty of evidence [12,13,14,25,40,41,43]. 

## 4. Discussion

The current umbrella review shows two main important facts. First, prevalence of non-adherence in LT patients can be high (especially among the younger adults), with a reported prevalence between 3% and 47%, yet in some reports it may reach up to 80% [12]. Second, there are many factors, not necessarily related to the medication, that can strongly influence non-adherence in LT adult and adolescent patients. Our systematic review was structured based on the WHO’s proposal regarding the predictors of adherence and non-adherence. The results of the review indicate that sociodemographic factors, factors related to the patient, factors related to the treatment, condition-related and health system-related factors are good categories of predictors for both adherence and non-adherence to immunosuppressive medication in LT patients.

We used the methodology of “umbrella review” and our bibliographic research covered a broad spectrum of the literature, having consulted four important databases, and finding a good number of reviews which satisfied the inclusion criteria. This methodology allowed us to update the existing information, including recent systematic reviews, and to describe in detail the evidence on factors related to treatment adherence after LT. After a comprehensive review of all the information, we were able to allocate the risk factors for non-adherence for LT patients into the five groups of factors related to non-adherence proposed by the WHO [31]. More importantly, the scope of this review includes risk factors for non-adherence not only in the adult population, but also in adolescents with LT, which are a high-risk population of non-adherence [45]. The review identified some second-level medication adherence factors. Those factors have shown an indirect association with medication non-adherence. 

The information provided by this study is intended to help the attending physicians to screen and detect non-adherence in LT patients. Nevertheless, a careful anamnesis including prior non-adherence, economical barriers to the therapy, eventual mental or addictive disorders and simplification of treatments (for example using extended release medications) especially in high-risk patients is fundamental. In this sense, it is important that pre-transplant assessments include the evaluation of medication adherence using validated questionnaires and dispensing records via electronic prescription. It is also essential, especially in liver transplantation due to possible previous addictions, to include a psychiatrist/psychologist in the liver transplant teams. The reviewed literature also discusses research needs and interventions to improve LT patient’s adherence, for example, highlighting the need for programs for adolescents to improve their medication self-management skills when transitioning to adults [38]. 

It is essential to identify candidates with ambivalence about treatment and prior history of non-adherence, substance abuse, poor social support, and poor organizational skills as they are more prone for treatment non-adherence [39]. Different authors revealed that simplifying treatment regimens is one of the most effective ways for improving adherence [12] or concluded that transplant programs must be furnished with sufficient resources and funding [42]. Early results suggest that more interventions with electronic monitoring, real-time adherence measurement and feedback, enhanced pharmaceutical care services, and targeted counselling are needed, as they have proven to be effective in this population [13]. With respect to electronic measurement systems, the promotion of personalized dosing systems for patients who require it is suggested, which can be carried out in pharmacies [46]. The importance of guidance through health education in relation to adherence to immunosuppressive therapy was also highlighted. The role of the nurse in the development of these activities is stressed for the promotion of safe behavior and the use of mechanisms that favor adherence in relation to immunosuppressive drugs [25]. 

It is important to address the cultural point of view that is not sufficiently analysed in the literature. As a result of limited access to deceased donors for cultural and religious reasons, Living Donor Liver Transplantation is the dominant approach in the Middle East and Asia. In adults, Living Donor Liver Transplantation offers advantages including superior outcomes and less resource utilization [47]. Although excellent results can be achieved after Deceased Donor Liver Transplantation in children, there is a clear benefit in the case of Living Donor Liver Transplantation in relation to patient and graft survival, even 1, 3 and 5 years after transplant. Transplantation with a shorter waiting time may prevent developmental impairment by reducing the frequency of hospitalization, progressive malnutrition, and growth retardation before LT, leading to better functional outcomes, especially among small children [48,49] This fact is important since poor adherence is associated with more years since the transplant [50]. 

In addition, the deficiencies in drug reimbursement or financials issues may increase the risk of non-adherence in live transplant patients. Potential socio-economic barriers such as insurance coverage and affordability of drug copays should be addressed prior to the transition. Serper et al. [13] examined financial barriers to transplant medication adherence by asking patients to report medication trade-offs, (e.g., choosing between food and essential medications). It would be good to know whether deficiencies in drug reimbursement programs may play a role in nonadherence, even among living donor recipients. This aspect should be considered in future studies [1,3].

This study has several limitations. First, the review studies included are not necessarily systematic reviews. Some were overviews or expert reviews. Only one study among the included reviews was a systematic review [44]. Second, some reviews were not purely focused on LT and included results on (non)adherence factors for other types of solid-organ transplantation. However, among those articles, only those containing a sufficient number of papers on LT were kept, to ensure consistency and comparability among all the reviewed texts. Third, although our review discovered many interesting factors for non-adherence of LT patients, aside from medication adherence factors, the rates of non-adherence are generally reported as an aggregate, and do not separate the non-adherence rates for missing appointments or drug holidays. Finally, the framework offered is general for any healthcare system, and therefore, specific countries would need to consider whether certain factors apply given the structure of the country’s healthcare system. Some factors, such as the high cost of medication, would only apply in countries where the treatment with immunosuppression is not covered by the healthcare system. Although it must also be considered that in some countries with public healthcare and coverage, there are patients who may find it difficult to pay for their medication each month since not everyone has 100% financing. All these limitations, then, are not in detriment to the quality of the work done, for according to the GRADE assessment, only articles showing a high or moderate certainty in their evidence presentation were kept for the analysis. 

Conducting studies to provide a better estimate of the adherence rates to medication, as well as tools (surveys or questionnaires) to gather evidence on non-adherence in this context and for other solid organ transplantation patients, along with programs to evaluate interventions to promote adherence in this context, are highly encouraged.

## 5. Conclusions

Prevalence of non-adherence in LT patients can reach levels of 80% (adolescents showing rates 4 times higher compared to adults), varying more often between 3–47%. A list of factors influencing non-adherence is proposed, based on the WHO classification of non-adherence (health system factors, socioeconomic factors, factors related to the treatment/therapy, patient related factors, and condition related factors). Each of these groups contains a subset of factors that have been proven to influence medication adherence after a LT, according to literature findings. This list of factors may help physicians to recognize patients with a potential risk of non-adherence and it could also lead to the designing of new tools to better understand non-adherence after LT and targeted interventions to promote adherence of LT patients. Conducting studies to provide a better estimate of the adherence rates to medication and behavioral recommendations, as well as tools to better understand the explanatory factors for non-adherence, along with programs to evaluate interventions to promote adherence in this context, are encouraged.

## Figures and Tables

**Table 1:** Results from the Umbrella Review on the identification of risk (enhancement) factors for non-adherence (adherence) of LT patients.

Reference, Country of Study, Type of Review, No. of Studies Included	Objective	Population/s Addressed	Adherence Measures Identified	Factors Associated with Non-Adherence/Adherence	Prevalence of Non-Adherence
Alonso et al. (2013) [38] USA and Canada Review/Expert Panel FLT Not specified but >10	To highlight priorities for clinical research that could successfully be conducted through the SPLIT collaborative and would have significant impact in pediatric liver transplantation.	Children (*) and Adolescent	Medication adherence. Degree of fluctuation (i.e., standard deviation, s.d.) of medication blood levels of tacrolimus in pediatric liver transplant recipients.	Developmental characteristics such as developing autonomy from family, assimilating with peers and separating from parents, poorly developed abstract thinking and understanding long-term consequences of present actions, are often difficult to balance with the behaviors required for optimal medication adherence. Self-management skills are integral to the achievement of independence necessary for successful healthcare transitions. Researchers and clinicians agree that adolescents and young adults should not transfer from pediatric to adult health services unless they have the skills necessary for functioning effectively in the adult healthcare system, including adhering to medication regimens.	5 to 80%, with adolescents having the highest rates of non-adherence
Anil Kumar & Mattoo (2015) [39] All countries (and India analyzed separately) Overview PLT A meta-analysis of 54 studies	To highlight the role of the psychiatrist and to sensitize the medical fraternity in general and the Organ Transplantation teams in particular.	All ages	Medication adherence and Behavioral. Medication adherence and substance abuse, measured by rates of alcohol relapses.	Patients with a prior history of alcohol abuse who use alcohol after liver transplantation are more likely to have problems of non-adherence to immunosuppressant medication. Though there is sufficient evidence to suggest post-OT substance use carries major adverse health consequences, a meta-analysis of 54 studies showed post liver OT relapse to any alcohol use twice more than relapse to heavy alcohol use (~6 vs. <3/100 ppy) and non-adherence to immunosuppressant medication being 3.2/100 ppy. These figures are comparable to cases with no prior history of abuse	3.2 per 100 persons per year
Bailey et al. (2021) [40] Countries unspecified (all) Review of Meta-analysis studies PLT 32 meta-analyses	To discuss the multiple aims of a psychosocial evaluation as part of the assessment for solid-organ transplantation, before providing an overview of both the required content and the practical process of undertaking such an assessment.	All ages	WHO definition for medication adherence is used. Subjective measurements of adherence involve a healthcare professional or patient’s self-reported evaluation of their behavior, often evaluated using a questionnaire. Objective measures of pre-transplant therapy adherence include (i) comparisons of the patient medication requests with prescribed use and predicted need for repeat dispensing (ii) assessments of the number/proportion of clinical appointments and treatment sessions missed, and (iii) observed adherence to a restriction or abstinence, for example evidence of alcohol or smoking avoidance, adherence to dietary and fluid restrictions.	Social support Liver transplant recipients with higher social support experienced higher odds of medication adherence post-transplant. The AASLD guidelines regarding liver transplantation state that a lack of social support is a contraindication to transplantation.	Not specified
Burra et al. (2011) [12] Countries specified (all) Review FLT 9 studies	This review analyzes the published literature on adherence in liver transplant patients with a particular focus on the reported prevalence of non-adherence and the identified risk factors.	Adult and Pediatric (*)	Evaluated areas of Non-adherence: Medication and behavioral. Clinical appointments, Medications (e.g., prednisolone), Immunosuppression, Relapse to alcohol/drug use.	For adult patients: High cost of medication, Young age (<40 years), Psychiatric disorders, Conviction that the medication is harmful, Side effects of medication.	3–47% (Non-adherence to medical regimens is reportedly nearly 4 times higher among pediatric and adolescent liver transplant patients versus adults)
Coilly et al. (2015) [41] France Review/Expert panel FLT Not specified	French experts in the liver transplantation field were asked to highlight pharmacokinetic (PK) differences between both formulations to assess efficacy and safety of the once a day formulation in the context of de novo initiation or conversion and to provide their recommendations for initiation and day-to-day management of Tacrolimus once a day formulation.	All ages	Medication Adherence to immunosuppressive therapy	Early conversion from tacrolimus twice a day to tacrolimus once a day	15–40% or 66.4% using VAS
Hammond et al. (2021) [42] Canada Brief review FLT Not specified but >15	To identify which patients will adhere to the more rigid aspects of post liver transplant care such as lifelong immunosuppressive medication adherence and a commitment to lifelong abstinence from alcohol, in cases where this is the precipitant for liver disease.	All ages	Medication adherence and Behavioral. Adherence to immunosuppressive medication and behaviors.	Missing clinical appointments Poor pre-operative adherence Socio-demographic factors: pediatric populations, unemployment at time of listing for liver transplant, males, having a pre-transplant diagnosis of mood disorder, being divorced High risk is associated with having 4–6 among the following factors (moderate risk 2–3 factors; low risk 0–1 factor): 1. DSM-IV compliant diagnosis of mood or anxiety disorder within the 24 months prior to transplant; 2. passive versus active coping styles in the face of emotional stressors; 3. presence or absence of documented medication adherence issues prior to transplant; 4. presence or absence of DSM-IV diagnostic criteria for substance abuse/dependence prior to transplant; 5. presence or absence of a primary caregiver as social support for the transplant recipient; and 6. the presence or absence of documented concerns regarding the stability of that social support system. Self-rate of the consequences of the transplant on patients’ life Having a lower perception about the necessity of medication (weaker beliefs that immunosuppressants could prevent rejection or that they were over prescribed by doctors) The number of immunosuppressants prescribed also reduced adherence rates (44.6% for 1 drug versus 32.2% for 2 drugs and 24.3% for 3 drugs; *p* = 0.02) Regarding alcohol abstinence: using active addiction treatment seems to moderate the recidivism rates of alcohol-related liver disease patients (improve compliance)	15–40% non-adherence rate for immunosuppressive medication
Jones & Serper (2020) [13] Europe and North America Overview FLT 6 studies	Reviews risk factors for non-adherence to medication of liver transplant patients as well as interventions to improve adherence of this population	Adult and adolescents	Adherence to medication and behavioral. Adherence to medication measured as deviation from the prescribed medication regimen sufficient to influence adversely the regimen’s intended effect, expanded by factors such as missed doses of medication, taking extra doses, drug holidays, variable timing of intake, and poor medication understanding. In this paper it is measured either from questionnaires (self-report) or electronic monitoring (recorded activity).	Unintentional non-adherence Higher number of comorbid conditions Medicare insurance Pre-transplant variables: longer time from transplant Psychosocial factors: poor social support, ongoing psychiatric illness, active substance abuse, low income or financial barriers, low literacy, lower educational attainment, and living alone. Medication-related factors: costs, medication side effects, regimen complexity, and lack of medication knowledge/poor medication understanding. Transition from adolescent age to adult age	6.7 per 100 persons per year
Kaplan et al. (2023) [14] Countries unspecified (all) Review FLT Not specified but >15	Understanding patient experience and the factors that contribute to it, including physical and psychological health, immunosuppression and medication adherence, return to employment or school, financial burden, and expectations, helps when thinking creatively about potential interventions to improve HRQOL.	Pediatric/adolescent recipients and living donor liver transplant (LDLT) recipients.	Adherence to immunosuppression (Definition of adherence may vary across studies)	Non-adherence to medication has been associated with both hospital readmission and increased healthcare costs. In one study, predictors of missing doses were male sex, longer time since transplant, pre-LT mood disorder, and pre-LT social support instability, while predictors of taking a different dose than prescribed were pre-LT mood disorder and pre-LT social support instability. Other factors that may impact adherence include treatment-related factors such as the cost and number of medications, the frequency with which medications are taken, and side effects associated with medications. Some pre-transplant factors to consider that may predict non-adherence include self-reported non-adherence before transplant, lower social support, higher education levels, and lower conscientiousness. Unemployment itself can lead to loss of insurance, financial difficulties and lower adherence to medication. Interventions targeted at adherence in the adolescent population: Adherence improved with the intervention.	Up to 45%
Ko et al. (2018) [43] Unspecified countries (all) Narrative Review FLT 11 studies	To synthesize the current findings and identify the gaps in knowledge about self-management in liver recipients. The specific aims of this study were to (1) identify areas of self-management that have been studied in liver recipients and (2) identify existing knowledge gaps regarding self-management in this population.	Adult recipients	Medication non-adherence and alcohol recidivism along with other self-management behaviors and activities such as clinic appointment attendance.	Demographic factors: younger, male, African American, employed, and divorced were more likely to be non-adherent. Transplant-related variables: Non-adherence more likely for recipients who had their transplants for a longer time period; recipients who reported more immunosuppressant-related symptom frequency and/or symptom distress; Recipients who did not keep clinic appointments, had negative perceptions of medication side effects, and low health literacy; Recipients who had intact perspective memory. Pre-transplant variables: Recipients who had a history of substance or alcohol abuse, medication non-adherence, and mental health needs before transplantation. Those who were unemployed at the time of listing and had limited social support before transplantation.	Reported rates of overall medication non-adherence, which included immunosuppressant taking, alteration, timing, and drug holiday, ranged from 39.4% to 66.4%. Medication non-adherence rates based on missing any doses were ranged from 8% to 62%. Timing non-adherence rates defined as not taking a dose at the prescribed time ranged from 27% to 64%, 25 altered dose rates ranged from 1% to 14%, 26 and drug holiday rates ranged from 0% to 39%. For studies using biochemical monitoring, 15% and 32% of participants appeared to be non-adherent, respectively, although different blood level criteria to determine medication non-adherence were used in two different studies.
Meng et al. (2019) [44] USA, Canada, Spain, Iran SLR and MA FLT 22 studies	To investigate such non-adherence after pediatric liver transplantation and risk factors associated with this non-adherence using findings of reported studies	Children (*) and adolescent	We examined three aspects of non-adherence outcomes: (1) immunosuppression medication non-adherence; (2) non-adherence to clinical attendance (patients do not follow doctor’s orders to regular clinic appointment and test, reflected from clinical record); (3) “global” non-adherence outcome (original author did not provide a specific non-adherence assessment aspect like immunosuppression or clinical attendance but could reflect non-adherence in multiple, global areas).	Older age of the pediatric patient, low family cohesion, poor social functioning, poor mental health and single-parent family.	The clinical attendance non-adherence rate was 45% (95% confidence interval [CI]: 39–51), global non-adherence rate was 17% (95% CI: 13–21) and immunosuppression non-adherence rates were 39% (95% CI: 26–52) and 34% (95% CI: 30–39) for cyclosporine and tacrolimus, respectively.
Oliveira et al. (2016) [25] North America and European Countries 191 studies	To investigate the evidence available in the literature on non-adherence to immunosuppressive therapy among patients undergoing liver transplantation	Adult	Adherence to immunosuppressive medication following liver transplantation	Risk factors related to the health service, such as control and reduction of the number of doses; Related to the individual, such as being male, divorced, alcohol or other substance user, exposed to low social support and being mentally ill.	Not provided

(*) Risk factors for children are not shown in this review. The paper included specific results on population other than children, and was, thus, included in the review. Abbreviations: FLT Fully focused on Liver Transplantation; PLT Multiorgan/Partially focused on Liver Transplantation; VAS Visual Analogue Scale.

**Table 2:** Factors identified for non-adherence among the reviewed reviews among WHO classification of factors for non-adherence.

Healthcare System	Socioeconomic Factors	Patient-Related	Treatment Related	Condition-Related
- Medicare insurance [13] - Lack of interventions targeted at adherence in the adolescent population (Adherence improved with the intervention) [14]	- Males [14,25,42,43] - Young age (<40 years) [12,43] - Older pediatric patients [44] - Pediatric populations [42] - African American [43] - Being divorced [25,42,43] - Poor social functioning or social support instability [13,14,25,40,43] - Low income or financial barriers [13] - Higher education levels before transplantation [14] - Low literacy barriers [13] - Low health literacy [43] - Employed [43] - Unemployment at time of listing for liver transplant (1–3) - Lower educational attainment [13] - Low family cohesion [44] - Single parent family [44] - Living alone [13] - Autonomy from family [38] - Poor abstract thinking [38] - Understanding long-term consequences of present actions [38] - Lower conscientiousness before transplant [14]	- Prior history of alcohol abuse reviews [39,43] - Missing clinical appointments [42,43] - Ongoing psychiatric illness [12,13] - Poor mental health [25,44] - Having a pretransplant diagnosis of mood disorder [14,42] - Mental health needs [43] - Active substance abuse [13,25] - Prior history of alcohol abuse [39,43] - Missing clinical appointments [42,43] - Prior history of medication non-adherence [43] - Self management skills: Ability for functioning effectively in the adult healthcare system (specially for adolescents) [38] - Having intact perspective memory [43] - Self-reported non-adherence before transplant [14] - Poor pre-operative adherence [42] - Conviction that the medication is harmful [12]	- Side effects [12,13,14,43] - High cost (1–3) - Difficult regimes [13,14,41] - Number of medications [14] - Lack of medication knowledge/poor medication understanding [13] - Lack of control and reduction of the number of doses [25] - More immunosuppressant related symptom frequency and/or symptom distress [43]	- Hospital readmission [14] - Higher number of comorbid conditions [13] - Longer time from transplant [13,14,43]

**Table 3:** Modified GRADE ratings for each study and the overall rating of strength of evidence.

Reference	Unified Statistical Analysis	Confounders Used	Outcomes of Interest Pre-Specified	Consistency of Results	Little Likelihood of Publication Bias	Large Sample	Certainty Evidence Rating
Alonso et al. (2013) [38]	1	1	1	1	1	1	High
Anil Kumar & Mattoo (2015) [39]	1	1	1	1	1	1	High
Bailey et al. (2021) [40]	1	0	1	1	1	1	Moderate
Burra et al. [12]	1	0	1	1	1	1	Moderate
Coilly et al. (2015) [41]	1	1	1	1	1	0	Moderate
Hammond et al. (2021) [42]	1	1	1	1	1	1	High
Jones & Serper (2020) [13]	1	1	1	1	1	0	Moderate
Kaplan et al. (2023) [14]	1	1	1	1	1	0	Moderate
Ko et al. (2018) [43]	1	0	1	1	1	1	Moderate
Meng et al. (2019) [44]	1	1	1	1	1	1	High
Oliveira et al. (2016) [25]	1	1	1	1	0	1	Moderate

Note: 1 if the criteria is fulfilled; 0 otherwise.

## Data Availability

Not applicable.

## Supplementary Materials

The following supporting information can be downloaded at: https://www.mdpi.com/article/10.3390/jcm13082348/s1, Table S1: Identified ideas for further research in the reviewed literature.
