# Supplementary material for: Risk Factors for Non-Adherence to Medication for Liver Transplant Patients: An Umbrella Review
Source: J Clin Med. 2024 Apr 18;13(8):2348. doi: 10.3390/jcm13082348 (PMC11051511; doi:10.3390/jcm13082348)
Supplement: Supplementary file 1 [file jcm-13-02348-s001.zip › jcm-2931058-supplementary.pdf]

Supplementary Table

Table S1. Identified ideas for further research in the reviewed literature.

Reference, Country of study, Type of review	Discussion on interventions, policies or further research needs to improve adherence of LT patients
Alonso et al (2013)<br>USA and Canada<br>Review/Expert Panel<br>FLT	General **on Adolescent issues.**<br>1 Assessment of the impact of executive functioning on medication adherence and transition readiness among adolescents.<br>Regarding **Transition Planning.**<br>1 There is a critical need for the development and validation of objective assessment tools to empirically evaluate the pediatric patient's readiness to move from a pediatric to adult-focused transplant health care. Transition readiness assessment tools should evaluate regimen knowledge, allocation of responsibility for healthcare tasks, self-management skills, and adherence.<br>2 Assessment of the impact of healthcare transition on measures of patient satisfaction, medical stability, quality of life, psychosocial functioning, educational/vocational outcomes, and healthcare utilization rates.<br>3 The timing of transfer from pediatric to adult-focused care should be individualized and based on the acquisition and mastery of self-management skills. Thus, research is needed to develop benchmarks to guide in determining the individualization of transfer to adult-centered care. The development of transition planning curricula and guidelines should include collaboration with the adolescent/young adult recipients and parents to determine how to best provide them with information related to the transition process.<br>4 Research is needed to define and identify predictors of successful transition to inform the development of programs which target modifiable factors. Research is needed to identify a standard transition practice for transplant providers to follow in order to enhance communication and collaboration between pediatric and adult caregivers during the transitional period.<br>5 Research investigating the role of health literacy and patient education in the transition process is also warranted.<br>**Specific Adherence promotion and self-management interventions.**<br>1 Future studies should continue to focus on developing a standardized method for routinely assessing medication adherence in pediatric liver transplant recipients.<br>2 The development of empirically based interventions to promote self-management skills is critical. Interventions should also target parental monitoring and the transition of responsibility of health-related tasks from the parent to the adolescent/young adult. Thus, interventions should focus on the role of parental monitoring and supervision of medication-related tasks as adolescents begin to demonstrate mastery of health management tasks.<br>3 Healthcare providers are responsible for the delivery of health-related information, fostering motivation, assisting their patients with the behavioral skills necessary for adherence, and collaborating on plans for chronic illness management. Future research should examine the impact of physician communication and motivational strategies on medication adherence in adolescents.<br>4 Interventions delivered using newer technologies, such as cell phone text messages and the internet, may be promising for promoting medication adherence in adolescents. Further investigation of the sustainability and effectiveness of these innovative eHealth interventions is needed.
Anil Kumar et al (2015)<br>All countries (and India analyzed separately)	It is very essential to **identify candidates with ambivalence about treatment and prior history of non-adherence, substance abuse, poor social support, and poor organisational skills** as they are more prone for treatment non-adherence.

Overview<br>PLT	
Bailey et al (2021)<br>Countries unspecified (all)<br>Review of Meta-analysis studies<br>PLT	Healthcare-related (rather than patient-related) factors are particularly understudied in the area of adherence-research. Adequately powered RCTs are required of interventions to improve adherence and psychosocial status pretransplant, to see whether these interventions can improve transplant access and outcomes for those with psychosocial challenges. Outcomes measured need to be those that matter to patients including graft health, life participation, life-expectancy, and likelihood of infection and cancer – rather than simply adherence or substance use.
Burra et al (2011)<br>Countries not specified (all)<br>Review<br>FLT	Simplifying the doses of immunosuppressive medications has recently emerged as one of the most important ways of improving adherence.
Coilly et al (2015)<br>France<br>Review/Expert panel<br>FLT	The experts consider adherence to treatment as a main advantage with the use of Tac qd, in day-to-day practice for LT patients.
Hammond et al (2021)<br>Canada<br>Brief review<br>FLT	As transplant programs come under increasing scrutiny to use their limited organ pool effectively and for maximal benefit, this must change. They in turn must be furnished with sufficient resources and funding to enable these challenges to be met effectively.
Jones et al (2020)<br>Europe and North America<br>Overview<br>FLT	To date, a handful of high-quality studies to improve medication adherence have been conducted among adult LT recipients. Early results suggest that high intensity, tailored, and multi-faceted interventions with electronic monitoring, real-time adherence measurement and feedback, enhanced pharmaceutical care services, and targeted counseling is more likely to be effective. However, more studies are clearly needed in this population. Future studies should further evaluate how technology-enabled solutions can be harnessed to promote adherence after LT.
Kaplan et al (2023)<br>Countries unspecified (all)<br>Review<br>FLT	Interventions aimed at improving physical and psychological functioning as well as medication adherence and reduction in hospitalisations have already been developed in both adult and pediatric/adolescent populations. More work is needed to develop transplant-specific quality of life measures and to intervene where deficits are found.
Ko et al (2018)<br>Unspecified countries (all)<br>Narrative Review<br>FLT	Future research efforts should focus on the comprehensive evaluation of self-management behaviors and activities beyond medication non-adherence and alcohol recidivism, consistent definitions of those behaviors and activities, and applying multiple measurements of those behaviors and activities (including subjective and objective measures). In addition, more efforts should be given to finding ways to improve self-management behaviors and activities. Randomized controlled trial design research is necessary to identify predictors of self-management behaviors and activities. Multiple research designs are also recommended as information gleaned from qualitative and quantitative studies will be beneficial for creating a more comprehensive picture of self-management and allow for more efficient intervention development.
Meng et al (2019)<br>USA, Canada, Spain, Iran<br>SLR and MA<br>FLT	Intervention based on risk factors for medical regimen non-adherence, such as mental health and family function, is necessary. In addition, a standard assessment of pediatric medical regimen non-adherence including as many dimensions as possible is required in order to be more objective and comprehensive when assessing non-adherence to medical regimen in pediatric liver transplantation.

Oliveira et al (2016)<br>North America and European Countries<br>Integrative Review<br>FLT	Therefore, the importance of proper guidance is mentioned, through health education in relation with adherence to immunosuppressive therapy. Thus, we highlight the role of the nurse in the development of these activities, promoting safe behavior and the use of mechanisms that favor adherence in relation to the immunosuppressive drugs.

Abbreviations: FLT Fully focused on Liver Transplantation; PLT Multiorgan/Partially focused on Liver Transplantation; VAS Visual Analogue Scale. (*) Risk factors for children are not showed in this review. The paper included specific results on population other than children, and was, thus, included in the review.
